# The Use of Social Media as a Persuasive Platform to Facilitate Nutrition and Health Behavior Change in Young Adults: Web-Based Conversation Study

**DOI:** 10.2196/28063

**Published:** 2022-05-18

**Authors:** Vanessa J Friedman, Cassandra J C Wright, Annika Molenaar, Tracy McCaffrey, Linda Brennan, Megan S C Lim

**Affiliations:** 1 Burnet Institute Melbourne Australia; 2 Monash University Melbourne Australia; 3 Menzies School of Health Research Darwin Australia; 4 Royal Melbourne Institute of Technology Melbourne Australia; 5 University of Melbourne Carlton Australia

**Keywords:** young adults, nutrition, physical activity, mental health, social media, qualitative methods, health promotion

## Abstract

**Background:**

Globally, suboptimal dietary choices are a leading cause of noncommunicable diseases. Evidence for effective interventions to address these behaviors, particularly in young adults, is limited. Given the substantial time young adults spend in using social media, there is interest in understanding the current and potential role of these platforms in shaping dietary behavior.

**Objective:**

This study aims to explore the influence of social media on young adults’ dietary behaviors.

**Methods:**

We recruited 234 young adults aged 18-24 years and living in Australia, using market and social research panels. We applied a digital ethnography approach to collect data from web-based conversations in a series of forums, where participants responded to different health-themed questions related to health behavior change and persuasion on social media. We conducted a qualitative thematic analysis.

**Results:**

Participants described how social media influenced their decisions to change their health behaviors. Access to social support and health information through web-based communities was juxtaposed with exposure to highly persuasive fast-food advertisements. Some participants expressed that exposure to web-based health-focused content induced feelings of guilt about their behavior, which was more prominent among women. Fast-food advertisements were discussed as a contributor to poor health behaviors and indicated as a major barrier to change.

**Conclusions:**

Young adults reported that social media is highly persuasive toward dietary behavior through different pathways of social influence. This suggests that social norms on the web are an important aspect of changing young adults’ health behaviors. The commercialization of social media also encourages poor health behaviors, largely through fast-food advertisements. Future social media–delivered dietary interventions should acknowledge the social and environmental factors that challenge the ability of young adults to make individual health behavior improvements. Care should also be taken to ensure that future interventions do not further elicit guilt in a way that contributes to poor mental health within this community.

## Introduction

### Young Adults’ Health and Nutrition

The prevalence of noncommunicable diseases is increasing globally, and they have become a leading health concern. It is known that suboptimal diets with low intake of fruits and vegetables and high intake of processed fatty foods are contributing to this trend [1]. Therefore, it is concerning that the diet quality of young adults typically reduces as they navigate the challenging shift from adolescence to adulthood [2]. Transitioning away from school and family resources toward workforce or further study has a strong and lasting impact on a young adult’s dietary behaviors [3,4]. Studies show that many young adults prioritize other aspects of their lives over healthy eating, which may be perceived as expensive and time-consuming for an age group that typically has low income [5,6]. The transitional nature of young adulthood can also present challenges in creating targeted and effective dietary interventions to reach this population [4]. Previous research has shown that young adults conceptualize health more broadly than physical health or the prevention of chronic disease and value mental, social, financial, and spiritual aspects [7]. As such, to develop engaging, feasible, and acceptable approaches that target dietary behaviors in this age group, the focus needs to extend beyond long-term health and should incorporate holistic views and short-term benefits [7].

Previous qualitative studies suggest that dietary behaviors during young adulthood are strongly influenced by internal perceptions and social norms [5,8,9]. Young adults were found to base their food choices on what they perceived their friends or family were eating, highlighting the power of socially normative messages in this domain [9]. Social media has become a key component of the social environment of young adults [8,10,11]. The ability to share, comment on, and react to other users’ posts increases interactions in this setting [12]. Social media delivers a constant stream of social input to young adults and has become a place for them to view and compare themselves with idealized versions of both their peers’ and strangers’ lives [7]. A recent systematic review indicated that image-related comparisons on social media may negatively impact the body image of young adults and drive poor eating behaviors such as restriction or overeating [13].

### Social Media and Nutrition

In 2018, a total of 99% of Australians aged 18-29 years used social media regularly, with 89% of them accessing their accounts at least once daily [14]. High rates of social media use have led food brands and companies to use social media to enhance their engagement with young adults [15]. Many fast-food companies use largely unregulated social media advertising regimes to promote energy-dense nutrient-poor foods that are shared throughout young adult peer networks [8,15]. Social media *influencers* have emerged as key players in these marketing strategies [16,17]. They are recognized as people who hold persuasive power by sharing their lives on various platforms and forming emotional connections with their audiences [16,17]. As such, companies work with influencers who provide paid product reviews to their audiences to boost the company’s sales and consumer engagement rates [17]. Some influencers exclusively post health and lifestyle content; however, many of these *health-focused influencers* lack professional accreditation and may post misleading nutrition advice that is not evidence-based [18]. Currently, experts in nutrition are becoming less trusted [19], and social media users are more likely to engage with and trust health-focused content from influencers than that from food industry or health promotion [20]. Hence, commentary from influencers has a relatively large impact on the values, beliefs, and behaviors of consumers regarding nutrition in both positive and negative ways [18].

The ability of social media to influence young adult audiences has also sparked interest from public health practitioners as a potential platform for health promotion [21]. Social media has previously been shown to influence health knowledge, with some studies identifying its positive influence on young adults through access to healthy recipes and exercises [10,11,22]. A recent systematic review identified that social media–delivered nutrition interventions that target adolescents and young adults lead to significant dietary improvements in 11 of 16 studies [11]. However, many of the interventions used were complex, with social media often being part of a secondary component, thus making it difficult to distinguish the true impact [11]. Our systematic review evaluated the efficacy of social media–delivered nutrition interventions in young adults only and identified that engagement with social media content varied greatly between 3% and 69% [22]. Young adults preferred to use social media passively, in a unilateral interaction, receiving information rather than sharing information [22]. Young adults were also not comfortable with talking about their weight on the web, highlighting the need to avoid weight-centric narrative in health promotion [22]. In 2 separate studies, we also found that social media users engage more frequently with food industry and lifestyle brands than with health promotion [20,23]. This highlights the need to develop more effective social media–delivered health promotion tools to encourage healthy behaviors in Australian young adults. Thus, the impact of social media on dietary behaviors must be further explored, and its persuasive abilities must be further understood.

### This Study

Phase 1a of the Communicating Health project seeks to gain insight into the use and application of social media, as it relates to 12 health-related and eating-related topics. This will allow for the identification of the channels, tones, and content-types that have the greatest potential for health promotion development. To understand how to develop effective social media–delivered health promotion tools, first, it is important to explore whether social media is currently impacting young adult dietary behaviors, and if so, how it is being used as a platform for persuasion. As such, this study aims to investigate what prompts young adults to make positive health and nutrition behavior changes and to understand the influence of social media as a persuasive medium on young adults’ health and nutrition behaviors.

## Methods

### Web-Based Conversations

This study is part of the larger Communicating Health project [24], which is a multistage mixed methods study that explores the dietary behaviors and social media use of Australian young adults. It aims to develop health promotion strategies using social marketing techniques. The data used in this study form a part of the formative phase of the Communicating Health project, phase 1a, which involved web-based conversations that explored young adults’ health, eating behaviors, and social media use [24]. An outline of all 4 phases of the Communicating Health project has been published previously [24]. The qualitative web-based conversations were hosted by an independent market research field house over a 4-week period. The web-based conversations were prompted by questions posed by the market research facilitators in moderated and secure web-based chat rooms. This method is based on digital ethnographic principles to understand how the digital aspects of society interact with the other material, sensory, and social aspects of human existence [25,26]. As a responsive data collection technique commonly used in consumer behavior research, web-based conversations allowed participants to interact with each other for a longer period than that allowed in traditional focus groups or interviews [27].

### Ethics Approval and Consent to Participate

Phase 1a received ethics approval from the RMIT Business College Human Ethics Advisory Network (project number 20489) and the Monash University Human Research Ethics Committee (project number 7807). Participants consented to anonymized findings being published when they completed the patient information and consent form before participating in the study. Ethics approval for this project was granted by the Monash University Human Research Ethics Committee (project ID 19417).

### Recruitment

Guided by previous studies with similar methodologies [28], the recruitment target was 200 young adults aged 18-24 years, living in Australia, and using social media at least twice a day. The recruitment period was from May 2017 to June 2017. This process was facilitated by an Australian Research Society–certified field house [29]. Young adults who had previously provided consent to participate in the research by signing up to market research panels were invited to participate in this study. Participants were from 3 research panels that were accredited by the International Organization for Standardization [30-32].

**Figure 1:**
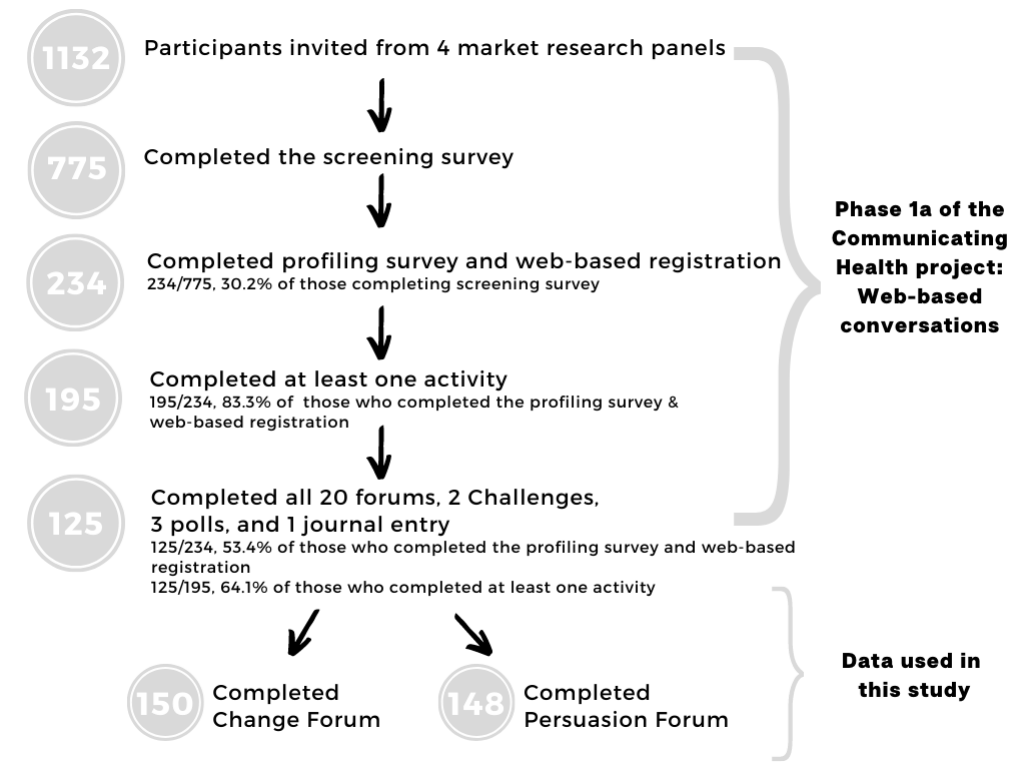
Participant flow diagram.

Panel members were sent an email invitation to complete a short screening questionnaire to assess their eligibility. Then, those who were eligible were asked to complete a profiling survey for collecting demographic information, self-reported weight and height (to calculate BMI [kg/m^2^]), social media use, and interest in health. Health interest (low or moderate and high) was determined by the median value of the following question asked in the profiling survey: “On a scale of 1-7 where 1 means ‘Strongly Disagree’ and 7 means ‘Strongly Agree’, please indicate how strongly you agree with the following statement - I take an active interest in my health.” The profiling survey was completed by 234 participants, who were then provided a link through email to sign up to the web-based conversation website. The participant flow diagram is shown in Figure 1. Then, the participants were stratified into 4 communities based on their age (18-21 years and 22-24 years) and interest in health (low or moderate and high). Those with low interest in health were grouped separately from those with moderate and high interest in health and, then, further divided by age, leading to 4 groups with 42-60 participants each. Profiling was set to achieve approximately equal number of participants in all groups and an approximately nationally representative distribution of gender and location (both Australian State or Territory and location type; ie, metropolitan and regional locations) [33]. All 4 communities had access to the same forums but could only interact with the members within their community. The dropout rate was high, which was expected for this age group. Therefore, a referral system was established, in which existing participants could refer a friend, who was then screened and profiled in the same way.

### Data Collection

The web-based conversations were conducted on a private web-based forum portal from May 10, 2017, to June 6, 2017. There were 20 forums in total (each took approximately 5 minutes to complete), 3 short polls, and an ongoing journal entry to which the participants were asked to contribute at least four times. The participants were also asked to complete 2 different challenges. The first challenge invited the participants to come up with a creative way to make more young adults to eat more fruits and vegetables. The second challenge asked the participants to persuade someone to kick-start a healthy lifestyle, using ≤160 characters. Then, the participants were able to see each other’s ideas and comment on their favorites. These different aspects of the web-based conversations explored different areas of health, nutrition, and social media, and participants responded to prompts from the market research moderators. The forums were released at different times but remained open for the 4-week period. Owing to different aspects being released on separate weeks, there were different numbers of participants who completed each forum (Figure 1). As per the standard practice by Australian Market and Social Research Society Limited, the participants were reimbursed for their time with a gift voucher worth Aus $100 (US $74.9) upon completion of all aspects of the web-based conversations, with a further Aus $100 (US $74.9) given to the 5 most descriptive and detailed forum responses from each web-based community (ie, 20 in total). This study reports on 2 of the forums that formed the web-based conversations: *catalysts for change* (referred to as *change*) and *persuasion on social media* (referred to as *persuasion*). These were chosen for analysis because they discussed health behavior change and persuasion on social media, which aligned with the research aim of this study. Table 1 describes the prompt questions used in both forums.

**Table 1:** Forum prompt questions.

Forum title	Discussion guide	Logic of enquiry
Catalysts for change	Have you changed anything recently to make you happier? Healthier? What triggered that change? How did you go about making that change? Did it change the dynamic within your friendship circle? Have you kept up with that change? (for how long – or probe for that?) What (if anything) was pulling you back to your old ways? Did social media give you any inspiration? Help? Hinder? Did anything else or any other tools play a role too (eg, apps, websites or even just people...)?	An exploration of what prompted lasting health behavior change in the young adult participants and whether social media played a role in this process.
Persuasion on social media	Can you think of times when you have used social media to persuade others to do something? Can you think of times when you have been persuaded? More broadly, can you think of how social media has influenced things you do in relation to health and healthy lifestyle?	To determine whether the participants viewed social media as a persuasive medium and to understand some of the factors that influenced its persuasiveness.

### Data Analysis

Descriptive analyses of the profiling survey data were conducted (Table 2). We undertook a thematic analysis of the forum responses using open coding and inductive techniques in alignment with a constructivist interpretation. As detailed in Table 3, this was guided by the systematic 6-step approach by Braun and Clarke [34]: data familiarization, generating initial codes, searching for themes, reviewing themes, defining and naming themes, and, finally, producing the report. This process enabled the large data set to be broken down into small units of comparison (codes) and rebuilt into themes that provided a systematic description of the participants’ experiences of health behavior change and social media use. A constant comparison approach was used to identify themes associated with healthy eating and other health behaviors [35]. Within this approach, each data point was compared with pre-existing data points to identify commonalities that existed within and between the participants’ responses. These commonalities became codes, and then, similar codes were grouped into broader themes that encapsulated the data set. Investigator triangulation was used to reduce subjectivity and enhance the rigor of the research findings [36]. As such, double-blind coding was conducted by 2 authors (VF and AM), who both coded each data set independently and, then, met to discuss their findings and reach an agreement. The researcher and primary coder of this study (VF) had a background in biomedical science and belonged to the age group of the participants. Growing up with social media, her empathetic connection to the study allowed her to extract details that may have otherwise been missed. The other coder (AM) had a disciplinary background in nutrition, which may have influenced her perception of the participants’ responses. The different backgrounds of each coder allowed the data to be examined from 2 different perspectives, which decreased subjectivity in the interpretation of the results. Although the forums occurred separately, data from both forums were analyzed together as overlapping themes were identified. Owing to different prompts in each forum (Table 1), some forums contributed more to certain themes than others.

**Table 2:** Demographic information of the participants who completed the change forum (n=150^a^).

Variable and category		Participants, n (%)
**Gender identity**		
	Women	87 (58)
	Men	62 (41.3)
	Nonbinary, gender-fluid, or genderqueer	1 (0.7)
**Age (years); health interest level (low or moderate and high)**		
	18-21; low	38 (25.3)
	18-21; moderate and high	47 (31.3)
	22-24; low	31 (20.7)
	22-24; moderate and high	34 (22.7)
**State**		
	Australian Capital Territory	1 (0.7)
	New South Wales	45 (30)
	Northern Territory	1 (0.7)
	Queensland	21 (14)
	South Australia	10 (6.7)
	Tasmania	4 (2.6)
	Victoria	42 (28)
	Western Australia	26 (17.3)
**Language spoken at home**		
	Culturally and linguistically diverse	42 (28)
	English	108 (72)
**BMI (kg/m^2^)**		
	<18.5 (underweight)	16 (10.7)
	18.5-24.9 (healthy weight)	82 (54.7)
	25-29.9 (overweight)	33 (22)
	≥30 (obese)	19 (12.7)
**Currently studying**		
	No	47 (31.3)
	Yes	103 (68.7)
**Current level of study**		
	High school (year 12)	7 (4.7)
	Technical and further education, college, or diploma	12 (8)
	University undergraduate course	75 (50)
	University postgraduate course	9 (6)

^a^Of the 150 participants who completed the change forum, 148 (98.7%) participants completed the persuasion forum.

**Table 3:** Approach to thematic analysis [34].

Step	Outline	How it applied to the analysis of both forums: catalysts for change (change) and persuasion on social media (persuasion)
1—Data familiarization	Full read through of the data set and noting emerging concepts and codes.	Each forum was read through twice. Emerging patterns and potential codes were noted for each.
2—Generating initial codes	Systematic identification and coding of relevant phenomena to generate a long list of codes.	A total of 3 rounds of coding were conducted for each forum. Round 1 Inductive line-by-line coding: each line of the data set was labeled based on its content, identifying novel, and expected codes. Similar codes were collapsed and redundant codes (ie, scarcely present in the data set) were deleted, resulting in 57 codes for the change forum and 76 codes for the persuasion forum. Round 2 Key elements were focused on (change: drivers of health behavior change; persuasion: how social media acted persuasively); emerging themes were noted. Codes were further collapsed, resulting in 54 codes for the change forum and 72 codes for the persuasion forum. VF and AM met to discuss the change forum—high level of agreement; more explicit coding was needed for health behaviors beyond nutrition (exercise, smoking, and alcohol) in VF’s codes. Round 3 Codes in the change forum were drawn out more distinctly, and coding for the persuasion forum focused on the impact of persuasion on health behaviors; emerging themes were noted. VF and AM met to discuss the persuasion forum and had complete agreement. Final collapsing and deleting resulted in 61 codes in each forum.
3—Searching for themes	Codes are compared and grouped into common themes. Considering the relationships between codes, emerging subthemes are generated.	Relationships between codes within each forum were considered to develop appropriate themes. Theme maps were generated in a hand-written format, using sticky notes to move codes around until they fit in the most logical sequence. These candidate themes were approved by AM with some slight adjustments to be made in the change forum.
4—Reviewing themes	All themes are reviewed for their relevance to the overall data set. Themes must be internally homogenous (contain similar codes) and externally heterogeneous (each theme is distinct).	Candidate themes were assessed for coherence (internal homogeneity) and distinction (external heterogeneity) in 2 steps: All extracts within each code were reviewed to ensure that they fit logically and were coherent. The data set was reread against the theme map to ensure that the themes were valid and representative of the overall data set. Any previously missed extracts were coded: change forum (2 new codes) and persuasion forum (0 new codes).
5—Defining and naming themes	The essence of what a theme captures within the data set is formed with textual evidence identified to portray each point. Each theme has a clear scope and succinct heading.	All themes were revised with textual evidence obtained from the data set to support each included code. Dot-point summaries were written for each theme and subtheme to capture their meaning, which were later developed into more comprehensive outlines. Theme and subtheme headings were developed. Throughout the process, a large degree of overlap emerged between the change and persuasion forums. As such, the themes were readjusted to combine the 2 analyses.
6—Producing the written report	The themes are collated into a written report that provides a description of the data set using extracts from the data set.	An integrated analysis of the change and persuasion forums was synthesized by incorporating textual evidence and written explanations to present the findings of the data set.

## Results

### Participant Characteristics

The characteristics of the participants based on self-reported data are presented in Table 2. Each forum had a different completion rate; *change* was completed by 76.9% (150/195), and *persuasion* was completed by 75.9% (148/195). Of the 150 respondents, most were women (n=87, 58%), reported moderate or high interest in health (n=81, 54%), had a healthy BMI (n=82, 54.7%), and were currently studying (n=103, 68.7%). Most participants lived in New South Wales (45/150, 30%) or Victoria (42/150, 28%) and spoke English at home (108/150, 72%).

### Thematic Analysis

#### Overview

Following a manual thematic analysis of both forums, the data sets were collated to develop 4 major themes from key recurring concepts. These included (1) peer support, (2) access to influencers and web-based communities, (3) advertising, and (4) constant exposure to content (Textbox 1).

**A brief description of the major themes from the data set.**

**Theme 1: peer support**

- Many participants described that social media provided them with access to support from their *real-world* peers who helped them to make and maintain a health behavior change. Alternatively, peer influence sometimes led to negative health behaviors such as buying fast food. Overall, participants simply wanted to be involved in what their peers were doing, whether this meant attending an exercise class together or buying fast food.

**Theme 2: access to influencers and web-based communities**

- Health-focused communities managed by persuasive social media influencers were considered by many participants as sources of support for making positive health behavior changes. Some participants believed that these communities enhanced their willpower, whereas other participants shared that they influenced their health attitudes but not their health behaviors. These communities also tended to promote an image-based perspective of health, which evoked feelings of guilt in some participants.

**Theme 3: advertising**

- Participants described social media newsfeeds that were flooded with advertisements, which they found persuasive. This included health products; however, fast-food advertisements had a more dominant presence. Promotions based on taste and affordability prompted fast-food purchases, which some participants viewed as lack of willpower.

**Theme 4: constant exposure to content**

- The design of social media to constantly expose its users to content was described as highly persuasive. Repeated exposure to health-themed content made the participants more conscious of their own health behaviors. Some participants explained that constantly viewing health content alongside fast-food advertisements made them feel conflicted and guilty if they consumed fast food.

#### Peer Support

The young adults in our study noted that their peers influenced their health behaviors through both direct communication on the web and exposure to the content they posted on social media feeds. A participant shared the following:

One of my friends would always message me to double check I was having breakfast and would always ask me what I had because she could tell when I was lying.

change forum; female; aged 18-21 years; low interest in health

Participants reported feeling influenced to make health behavior improvements when their peers posted on social media about their own positive changes. This was exemplified by a participant who wrote the following:

I also sometimes get influenced to be fitter when people share on social media their own health transformations. I then reconsider my lifestyle and see what I can do to be more active and healthy myself.

persuasion forum; male; aged 18-21 years; low interest in health

Many participants shared that peer influence on social media drove both positive and negative health behaviors based on the content being shared:

Knowing what my friends eat...can encourage me to eat certain things. When a person shares an exercise [post], I am more inclined myself to exercise...when a friend makes a comment on a [Fast food brand name removed]’s post, I am more inclined to check out their deals.

persuasion forum; male; aged 22-24 years; moderate and high interest in health

Some participants, most of whom were men, also shared their health activities with peers on social media to reach out for support and hold themselves accountable. A participant explained the following:

To put myself out there on social media really gave me the confidence and gumption to stick to my routine.

persuasion forum; male; aged 22-24 years; low interest in health

#### Access to Influencers and Web-Based Communities

In addition to receiving peer support from friends or acquaintances, the participants also experienced community support through health-focused pages or communities on social media, which were often managed by health-focused influencers. These communities provided participants with access to useful diet and lifestyle information and motivated them by providing a sense of unity and connection with others. A participant shared the following:

Social media has had an extremely positive influence on me when it comes to maintaining a healthy lifestyle...It’s 24/7 access to help, reassurance and motivation.

persuasion forum; female; aged 22-24 years; moderate and high interest in health

Female participants with moderate and high interest in health more commonly discussed social media as a positive influence.

The participants also described willpower as an important moderator of the influence that social media content had on their behavior. A participant believed the following:

If I try hard enough to work on my eating and doing more exercise then I will be able to be like them [models] with their hundreds of likes on their photos.

persuasion forum; male; aged 18-21 years; moderate and high interest in health

Web-based health communities could motivate these highly driven participants to remain self-disciplined and self-reliant, which helped them to resist negative external influences such as fast-food advertisements. A participant explained the following:

I find it’s easier to stay motivated if I stay home and in routine (without access to bad food of course), and interact regularly with the online fitness/health community.

change forum; female; aged 22-24 years; moderate and high interest in health

This was most often discussed by participants aged 18-21 years, with moderate and high interest in health. In contrast, some participants revealed that health-focused communities influenced their attitudes toward health, but not always their behaviors. A participant shared the following:

I’m following many health and ‘fitspo’ blogs and pages which teach me simple recipes and exercise regimes- now whether I actually follow them or not is another question.

persuasion forum; female; aged 18-21 years; low interest in health

This concept was most often described by women aged 18-21 years, with low interest in health.

A disadvantage of social media health-focused communities described by some participants was their tendency to portray health in an “image-fueled way” (*persuasion* forum; female; aged 18-21 years; moderate and high interest in health). For some participants, this led to feelings of self-doubt from upward comparisons with photos of others on the web. This was exemplified by a participant who wrote the following:

Seeing health/fit looking people on social media...can either inspire people to be healthier or they can discourage people as their body/lifestyle/look is unattainable.

persuasion forum; female; aged 18-21 years; moderate and high interest in health

Participants aged 18-21 years more readily associated guilt with health content on social media. The female participants were largely discouraged by this guilt, whereas the male participants discussed that upward comparisons motivated them to make a change.

Other participants described an awareness that their repeated exposure to social media health-focused influencers affected their outlook on which health behaviors they adopted:

While I love hiking and outdoors activities i’m not sure whether that’s entirely due to my own interests or because I see social media influencers with the perfect life doing things like that too...maybe I feel like that’s what having a healthy balanced life is like because that’s how it’s portrayed on social media.

persuasion forum; female; aged 22-24 years; low interest in health

Some participants also expressed indifference or disinterest toward social media in general. A participant stated the following:

Social media hasn’t really changed anything, because I don’t really like sharing my thoughts and activities through social media.

change forum; male; aged 22-24 years; moderate and high interest in health

This viewpoint was more commonly described by male participants. Similarly, most participants did not engage with social media as a means to actively interact with others, but used it as a purely observational platform. This was most commonly observed in those participants who were aged 22-24 years, with moderate or high interest in health.

#### Advertising

Participants reported that advertisements on social media also had an impact on their drive to make a health behavior change. Many participants described being drawn to health products that advertised benefits such as weight loss, feeling better, or affordability. A participant was allured by a detox tea, as it claimed “to help prevent bloating, slim your tummy and give you extra vitamins*”* (*persuasion* forum; female; aged 18-21 years; low interest in health). Participants aged 18-21 years, with low interest in health, more often discussed the persuasive effects of social media advertisements. Exposure to advertisements from large corporations that appeared *randomly* on participants’ newsfeeds were more often discussed than paid influencer content or products.

Fast-food advertisements were described as having a dominant presence on social media newsfeeds, which influenced the participants’ food choices. A participant explained the following:

Most ads on Facebook influence my health negatively...as they are usually for unhealthy food options.

persuasion forum; male; aged 22-24 years; low interest in health

This notion was discussed more often by female participants and those aged 18-21 years. Male participants more commonly referred to cost-based advertisements of fast food as persuasive:

These [fast-food] meals are cheap and easy, [and] although they’re[sic] aren’t healthy I know they will taste good. This [fast-food] advertising is very persuasive as it makes me believe that I am hungry and I can not[sic] stop thinking about the new promotion.

persuasion forum; male; aged 18-21 years; moderate and high interest in health

Some participants who revealed the difficulties in resisting fast-food advertisements viewed their temptation as a lapse in self-discipline. A participant remarked the following:

I may have been ‘persuaded’ (read ‘reminded of my weak will’) to purchase [fast food brand name removed] on several occasions.

persuasion forum; male; aged 18-21 years; low interest in health

#### Constant Exposure to Content

The participants described that being frequently exposed to health-themed or food-themed content was a highly influential aspect of social media. They believed that the way content was presented on social media was more persuasive than the content itself, explaining that they were more likely to engage with something if it appeared frequently in their newsfeeds. This was exemplified by a participant who shared the following:

While social media can be used as a platform...to persuade, I really thing [sic] social media...does most of the persuading [itself].

persuasion forum; female; aged 18-21 years; moderate and high interest in health

Although this was discussed in reference to fast-food advertisements, it was more often applied to health content, which encouraged some participants to be more health-conscious:

I see a lot about healthy lifestyle and fitness in my social media feeds and I think that constant exposure has made me much more conscious of the choice I make, and a bit more aware of exercising and eating healthy.

persuasion forum; female; aged 22-24 years; low interest in health

Although constant exposure to both general and health-themed content increased engagement in healthy behaviors in most participants, a participant described that they “didn’t want to engage in something that was being shoved in my face every time I opened Facebook, Twitter, and even Instagram” (persuasion forum; female; aged 18-21 years; low interest in health).

Some participants described that the cohabitation of health content and food temptation on the web made them feel guilty about their health behaviors. Their repeated exposure to these conflicting health ideals placed side-by-side evoked poor mental health and body image:

In relation to health and lifestyle it [social media] has not at all helped because it always shows videos of tasty unhealthy recipes and ads for [Fast food brand name removed] and [Fast food brand name removed]...It also then shows me photos of tall, tan, skinny models which makes me feel so bad about eating all the fast food.

persuasion forum; female; aged 18-21 years; low interest in health

## Discussion

### Principal Findings

This study aimed to contribute to a growing body of research that defines the interplay between young adults’ health behaviors and social media. Specifically, this study aimed to address current gaps regarding what prompts young adults to make positive health and nutrition behavior changes and to understand how social media acts as a platform for persuasion in this process. Our study found that peer support was crucial in shaping young adults’ health behaviors and that using social media to both message friends and view their posts prompted change. Web-based health-focused communities were also identified as a source of support, and health-focused influencers at their helm were found to play a prominent role in persuasion. Other persuasive aspects of social media included fast-food advertisements and constant exposure to content through newsfeeds. These aspects influenced participants’ health behaviors, particularly regarding purchasing fast food or being more conscious of dietary choices.

The participants of this study highlighted social influence as a key driver of health behavior change in social media–based peer interactions. The significance of real-world peer influence on young adults’ health behavior change is well established in the literature [37-39]. The results of this study suggest that young adults also find valuable social support in web-based environments. For example, seeing peers posting about their own healthy behaviors inspired some participants to follow their lead. In contrast, participants were also persuaded to purchase fast food if their friends were sharing posts from these brands. As such, this study indicates that young adults are likely to align their health behaviors with the actions of their peers, regardless of whether it is a positive or negative action. These findings are supported by social cognitive theory, which posits that people will mimic their peers to gain social acceptance [40]. Moving forward, targeting peer networks rather than individuals may enhance social media–delivered health promotion techniques. For this to be done effectively, further research may be needed to gain a greater understanding of how peer networks communicate on social media.

In addition to peers, the participants also identified health-focused influencers and web-based communities as having persuasive power over their dietary behaviors. Past research indicates that lifestyle brands, including influencer pages, on Facebook and Instagram have higher levels of engagement than both food industry and health promotion pages [20,23]. Their engagement is likely increased by their use of relatable content, positive emotional messages, paid promotions, and simple diet and exercise tricks that promise happiness by achieving appearance-related goals [20,23,41]. Our results moderately support this narrative, as some participants discussed influencers, particularly health-focused influencers, as a source of motivation to make and maintain positive health behavior change. However, some participants also explained that content from health-focused influencers only altered their attitudes toward health and did not lead to tangible behavior changes. Moreover, advertisements from large companies were discussed more often as a source of persuasion than influencers or influencer-promoted products in this study. These issues have recently been exemplified in the *Girls Make Your Move* campaign, which received funding from the Australian Department of Health to increase the involvement of girls aged 12-21 years in sport [42]. Although influencers were involved in the social media promotion of this campaign, other techniques such as viewing advertisements on YouTube or interacting with campaign posts on social media platforms led to more tangible behavior changes [42]. Moreover, the Australian Federal Health Minister recently launched an investigation into the campaign after learning that some of the influencers involved were also sponsored by alcohol brands and displayed racist or homophobic behavior on the web [43]. Moving forward, public health organizations need to remain cautious about engaging with influencers on social media for health promotion. Furthermore, our study suggests that additional research may be needed to determine the extent of influence caused by influencers, regarding young adults’ health behavior change, particularly when competing with mainstream brands for attention.

Social media environments have become heavily commercialized, and many companies pay for greater exposure to maximize their reach among young adults [44,45]. Advertisements delivered on social media is poorly regulated compared with traditional advertisements, making young users increasingly vulnerable to the persuasive tactics used by large corporations [46]. The participants of this study discussed that viewing fast-food advertisements on social media often led them to purchase fast food. The social media newsfeeds are designed such that the participants were constantly exposed to this content, which they found to be a key aspect of their persuasive abilities. Research has demonstrated that passively receiving advertisements on social media increases brand engagement and product sales, even if consumers did not have interest in the product initially [8,47]. Our study also showed that exposure to health-focused content in this manner led participants to be more aware of their health behaviors. It could be deduced that it was not the content that each individual was viewing that was persuasive, but their repeated exposure to it. The content that an individual views on their social media feeds is curated by an algorithm that predicts their likes, interests, and needs based on their behavior on the web [48], which leads to the creation of echo chambers [49]. Consequently, the more often an individual or their peers engage with social media–delivered fast-food advertisements, the more often they will be shown this content. In contrast, if an individual engages with health-focused content more regularly, this content will be displayed for them more often, which can lead to more positive health behaviors. A key issue for future social media–delivered health promotion to overcome will be ensuring that individuals with low interest in health also receive important health information that otherwise may not be *selected* for them in their echo chambers, owing to their patterns of behavior on the web.

Regardless of the heavily commercialized and persuasive setting of social media, another finding of this study was that the participants still viewed their health behaviors as an individual responsibility. This was encapsulated in the participants’ beliefs that their ability to achieve a healthy lifestyle as shown to them by health-focused influencers was solely dependent on their work ethics and willpower. As described by a participant, “giving in to the temptation” of fast-food advertisements was viewed as an indicator of their own weak will, rather than the persuasive tactics used by the fast-food brand. This outlook is well documented in the literature and indicates an association of moral values with an ability to practice positive health behaviors [50-53]. Instead of approaching healthy lifestyles from this neoliberal meritocratic perspective, creating a more health-promoting environment on social media may garner greater community awareness of and involvement in healthy behaviors [37,39,54]. A way to achieve this may be to introduce regulation around social media–delivered advertising campaigns, such as limiting the number of times fast-food advertisements can appear on an individual’s newsfeed or using fact-checking systems for health-related posts. Policy reforms regarding social media may also help health promotion to reach a wider range of consumers.

Another key finding of this study was the association of guilt with content from health-focused communities on social media, which is well established in the literature [10,18,55,56]. Young adults are increasingly looking toward health-focused communities for diet and lifestyle guidance, which can have serious consequences related to mental health and body image [10,18,55-58]. They often place greater value on appearance than on health and idealize lean physiques formed through restrictive diet and exercise regimes [55,58,59]. Research indicates that visual comparisons with these body ideals can be detrimental to young adults’ self-image and lead to poor mental health [13,55,59-61]. Other studies, including the systematic literature review by Rounsefell et al [13], indicate a link between these comparisons and disordered eating behaviors such as dieting or restricting food and overeating [18]. Participants in this study shared their own feelings of guilt when they were unable to follow the advice of health-focused influencers or achieve their health goals. The coexistence of health-focused content with fast-food advertisements on participants’ newsfeeds only exacerbated this condition. Previous findings from our Communicating Health project indicate a moral association with dietary behaviors [53]. In combination with this study, these findings suggest that people may perceive health-focused influencers as the angel on one shoulder and fast-food advertisements as the devil on the other shoulder. Those who follow health-focused pages are shown a message that makes them believe that it is more moral to practice healthy behaviors. When they are unable to follow through with these behaviors, for example, owing to the persuasive impact of fast-food advertisements, this may lead to cognitive dissonance and guilt, as seen in some of our participants.

This study also indicated a gendered response to guilt from viewing health content on social media. Female participants more often discussed the detrimental effects of health content and felt discouraged by upward comparisons, whereas male participants found these to be motivational. These findings contribute to an emerging conversation regarding the impact of health-focused social media content on different genders [18,62,63]. Women are often perceived to be more vulnerable to the negative impacts of health-focused content on social media, as our own study indicates, and have previously been shown to access diet-related and exercise-related social media posts more commonly than men [64]. However, a growing body of literature suggests that these notions may be caused by gender norms that reduce the likelihood of men openly sharing their experiences with negative body image [18,62,63]. Male participants in the qualitative study by Easton et al [18] revealed negative impacts similar to those experienced by women, a pattern further indicated by a recent cross-sectional survey by DiBisceglie et al [62]. Moreover, a recent study identified that men were featured and objectified in health and fitness content on social media almost as often as women [65]. Further research is needed to clarify whether a meaningful gender-based difference exists in the way that web-based health-focused content is consumed. However, care should be taken to ensure that future social media–delivered health interventions avoid appearance-based health messages to protect young adults’ mental health.

### Limitations

This study had some limitations. As our data collection was completed during the examination period of Australian Universities, challenges regarding recruitment and participant dropout emerged. Consequently, our participants may not be generally representative of the Australian population. Our sample also included more women than men and a large proportion of students and young adults who were well educated. Our analysis technique included searching for commonalities among the data, which may mean that the experiences of women and students were captured more strongly than those of others. However, we also spent time in contrasting discrepant cases to ensure that less common but still important themes were captured. The conversational design of the forum also may have introduced groupthink, social comparison bias, and recall bias. The dropout rate and different numbers of participants completing each forum may indicate participant fatigue in completing the web-based conversations over an extended period. Moreover, participants may have defined positive or negative health behaviors differently from each other owing to the subjective nature of the topic. Finally, this study was conducted at a particular time with a particular group of Australian young adults. Social media, among other technologies, evolves rapidly. Hence, further research will be necessary as the platforms grow and change.

### Conclusions

This study contributed to a greater understanding of the role of social media in health behavior change among young adults. Social factors play a key role in prompting positive health behavior changes. Future studies should develop a greater understanding of social interactions and peer networks in a web-based environment to guide the development of integrated health promotion techniques. The persuasive effect of social media on participants’ health behaviors was largely attributable to advertisements and constant exposure to content. This study suggests that young adults view health as an individual responsibility and place great value on self-discipline. A shift toward minimizing external pressures through policy changes and regulation of advertisements needs to be encouraged. Policy reform may also assist health promotion in reaching social media users who are disinterested in health. Finally, future social media–delivered health interventions need to be mindfully developed to ensure that they do not further elicit guilt among social media users.

## References

[1] (2013). Global action plan for the prevention and control of NCDs 2013-2020. World Health Organization.

[2] (2017). National health survey: first results, 2017-18 financial year. Australian Bureau of Statistics.

[3] Winpenny EM, van Sluijs EM, White M, Klepp KI, Wold B, Lien N (2018). Changes in diet through adolescence and early adulthood: longitudinal trajectories and association with key life transitions. Int J Behav Nutr Phys Act.

[4] Arnett JJ (2000). Emerging adulthood. A theory of development from the late teens through the twenties. Am Psychol.

[5] Deliens T, Clarys P, De Bourdeaudhuij I, Deforche B (2014). Determinants of eating behaviour in university students: a qualitative study using focus group discussions. BMC Public Health.

[6] Cha E, Crowe JM, Braxter BJ, Jennings BM (2016). Understanding how overweight and obese emerging adults make lifestyle choices. J Pediatr Nurs.

[7] Molenaar A, Choi TS, Brennan L, Reid M, Lim MS, Truby H, McCaffrey TA (2020). Language of health of young Australian adults: a qualitative exploration of perceptions of health, wellbeing and health promotion via online conversations. Nutrients.

[8] Howse E, Hankey C, Allman-Farinelli M, Bauman A, Freeman B (2018). 'Buying salad is a lot more expensive than going to McDonalds': young adults' views about what influences their food choices. Nutrients.

[9] Pelletier JE, Graham DJ, Laska MN (2014). Social norms and dietary behaviors among young adults. Am J Health Behav.

[10] Vaterlaus JM, Patten EV, Roche C, Young JA (2015). #Gettinghealthy: the perceived influence of social media on young adult health behaviors. Comput Human Behav.

[11] Chau MM, Burgermaster M, Mamykina L (2018). The use of social media in nutrition interventions for adolescents and young adults-a systematic review. Int J Med Inform.

[12] Papasolomou I, Melanthiou Y (2012). Social media: marketing public relations’ new best friend. J Promot Manag.

[13] Rounsefell K, Gibson S, McLean S, Blair M, Molenaar A, Brennan L, Truby H, McCaffrey TA (2020). Social media, body image and food choices in healthy young adults: a mixed methods systematic review. Nutr Diet.

[14] (2018). Yellow social media report 2018. Part one - consumers. Yellow.

[15] Freeman B, Kelly B, Vandevijvere S, Baur L (2016). Young adults: beloved by food and drink marketers and forgotten by public health?. Health Promot Int.

[16] Freberg K, Graham K, McGaughey K, Freberg LA (2011). Who are the social media influencers? A study of public perceptions of personality. Public Relat Rev.

[17] Uzunoğlu E, Misci Kip S (2014). Brand communication through digital influencers: leveraging blogger engagement. Int J Inf Manag.

[18] Easton S, Morton K, Tappy Z, Francis D, Dennison L (2018). Young people's experiences of viewing the fitspiration social media trend: qualitative study. J Med Internet Res.

[19] Penders B (2018). Why public dismissal of nutrition science makes sense: post-truth, public accountability and dietary credibility. Br Food J.

[20] Klassen KM, Borleis ES, Brennan L, Reid M, McCaffrey TA, Lim MS (2018). What people "Like": analysis of social media strategies used by food industry brands, lifestyle brands, and health promotion organizations on Facebook and Instagram. J Med Internet Res.

[21] Moorhead SA, Hazlett DE, Harrison L, Carroll JK, Irwin A, Hoving C (2013). A new dimension of health care: systematic review of the uses, benefits, and limitations of social media for health communication. J Med Internet Res.

[22] Klassen KM, Douglass CH, Brennan L, Truby H, Lim MS (2018). Social media use for nutrition outcomes in young adults: a mixed-methods systematic review. Int J Behav Nutr Phys Act.

[23] Barklamb AM, Molenaar A, Brennan L, Evans S, Choong J, Herron E, Reid M, McCaffrey TA (2020). Learning the language of social media: a comparison of engagement metrics and social media strategies used by food and nutrition-related social media accounts. Nutrients.

[24] Lombard C, Brennan L, Reid M, Klassen KM, Palermo C, Walker T, Lim MS, Dean M, Mccaffrey TA, Truby H (2018). Communicating health-optimising young adults' engagement with health messages using social media: study protocol. Nutr Diet.

[25] Pink S, Horst H, Postill J, Hjorth L, Lewis T, Tacchi J (2016). Digital Ethnography: Principles and Practice.

[26] Brennan L, Fry ML, Previte J (2015). Strengthening social marketing research: harnessing “insight” through ethnography. Australas Mark J.

[27] Dessart L, Veloutsou C, Morgan-Thomas A (2015). Consumer engagement in online brand communities: a social media perspective. J Prod Brand Manag.

[28] (2013). Drinking-related lifestyles: exploring the role of alcohol in Victorians' lives. VicHealth.

[29] (2017). Code of professional behaviour. The Research Society.

[30] (2019). In the world of data.. scale matters accuracy matters. Dynata.

[31] (2019). Australian federal election: it’s getting political!. Pureprofile.

[32] (2017). Our research services. Student Edge.

[33] (2016). 3101.0 - Australian Demographic Statistics, Jun 2016. Australian Bureau of Statistics.

[34] Braun V, Clarke V (2006). Using thematic analysis in psychology. Qual Res Psychol.

[35] Lewis-Beck MS, Bryman A, Futing Liao T (2004). The SAGE Encyclopedia of Social Science Research Methods.

[36] Patton MQ (2014). Qualitative Research & Evaluation Methods: Integrating Theory and Practice. 4th edition.

[37] Strong KA, Parks SL, Anderson E, Winett R, Davy BM (2008). Weight gain prevention: identifying theory-based targets for health behavior change in young adults. J Am Diet Assoc.

[38] Grunseit AC, Cook AS, Conti J, Gwizd M, Allman-Farinelli M (2019). "Doing a good thing for myself": a qualitative study of young adults' strategies for reducing takeaway food consumption. BMC Public Health.

[39] Stok FM, de Vet E, de Ridder DT, de Wit JB (2016). The potential of peer social norms to shape food intake in adolescents and young adults: a systematic review of effects and moderators. Health Psychol Rev.

[40] Munt AE, Partridge SR, Allman-Farinelli M (2017). The barriers and enablers of healthy eating among young adults: a missing piece of the obesity puzzle: a scoping review. Obes Rev.

[41] Pilgrim K, Bohnet-Joschko S (2019). Selling health and happiness how influencers communicate on Instagram about dieting and exercise: mixed methods research. BMC Public Health.

[42] (2019). Girls make your move: campaign evaluation research. Department of Health, Australian Government.

[43] Sweeney L (2018). Health Minister announces urgent investigation into taxpayer-funded campaign working with Instagram influencers. ABC News.

[44] Holmberg C, E Chaplin JE, Hillman T, Berg C (2016). Adolescents' presentation of food in social media: an explorative study. Appetite.

[45] Freeman B, Kelly B, Baur L, Chapman K, Chapman S, Gill T, King L (2014). Digital junk: food and beverage marketing on Facebook. Am J Public Health.

[46] Montgomery KC, Grier SA, Chester J, Dorfman L, Williams JD, Pasch KE, Collins CA (2013). The digital food marketing landscape: challenges for researchers. Advances in Communication Research to Reduce Childhood Obesity.

[47] Naylor RW, Lamberton CP, West PM (2012). Beyond the “Like” button: the impact of mere virtual presence on brand evaluations and purchase intentions in social media settings. J Mark.

[48] Eslami M, Rickman A, Vaccaro K, Aleyasen A, Vuong A, Karahalios K, Hamilton K, Sandvig C (2015). "I always assumed that I wasn't really that close to [her]": reasoning about invisible algorithms in news feeds. Proceedings of the 33rd Annual ACM Conference on Human Factors in Computing Systems.

[49] Parker L, Brennan L (2020). Social Marketing and Advertising in the Age of Social Media.

[50] Coffey J (2014). ‘As long as I’m fit and a healthy weight, I don’t feel bad’: exploring body work and health through the concept of ‘affect’. J Sociol.

[51] Coffey J, Kelly P, Pike J (2017). 3 Youth, health and morality: body work and health assemblages. Neo-Liberalism and Austerity: The Moral Economies of Young People’s Health and Well-Being.

[52] Moore SE (2010). Is the healthy body gendered? Toward a feminist critique of the new paradigm of health. Body Soc.

[53] Brennan L, Klassen K, Weng E, Chin S, Molenaar A, Reid M, Truby H, McCaffrey TA (2020). A social marketing perspective of young adults' concepts of eating for health: is it a question of morality?. Int J Behav Nutr Phys Act.

[54] Kuipers G (2019). Cultural narratives and their social supports, or: sociology as a team sport. Br J Sociol.

[55] Raggatt M, Wright CJ, Carrotte E, Jenkinson R, Mulgrew K, Prichard I, Lim MS (2018). "I aspire to look and feel healthy like the posts convey": engagement with fitness inspiration on social media and perceptions of its influence on health and wellbeing. BMC Public Health.

[56] Perloff RM (2014). Social media effects on young women’s body image concerns: theoretical perspectives and an agenda for research. Sex Roles.

[57] Tiggemann M, Zaccardo M (2015). "Exercise to be fit, not skinny": the effect of fitspiration imagery on women's body image. Body Image.

[58] Jong ST, Drummond MJ (2016). Exploring online fitness culture and young females. Leis Stud.

[59] Boepple L, Ata RN, Rum R, Thompson JK (2016). Strong is the new skinny: a content analysis of fitspiration websites. Body Image.

[60] Want SC (2009). Meta-analytic moderators of experimental exposure to media portrayals of women on female appearance satisfaction: social comparisons as automatic processes. Body Image.

[61] Brannan ME, Petrie TA (2008). Moderators of the body dissatisfaction-eating disorder symptomatology relationship: replication and extension. J Couns Psychol.

[62] DiBisceglie S, Arigo D (2021). Perceptions of #fitspiration activity on Instagram: patterns of use, response, and preferences among fitstagrammers and followers. J Health Psychol.

[63] Palmer L (2015). ‘‘Poppin’ bottles, getting wheysted.” Exploring young men’s engagement with fitspiration content and its consequential influences on attitudes and behaviour. J Promot Commun.

[64] Carrotte ER, Vella AM, Lim MS (2015). Predictors of "Liking" three types of health and fitness-related content on social media: a cross-sectional study. J Med Internet Res.

[65] Carrotte ER, Prichard I, Lim MS (2017). "Fitspiration" on social media: a content analysis of gendered images. J Med Internet Res.

